# Inhibition of HIF-1α-AQP4 axis ameliorates brain edema and neurological functional deficits in a rat controlled cortical injury (CCI) model

**DOI:** 10.1038/s41598-022-06773-9

**Published:** 2022-02-17

**Authors:** Ao Xiong, Junxia Li, Renping Xiong, Yiming Xia, Xu Jiang, Fuyang Cao, Hong Lu, Jianzhong Xu, Fabo Shan

**Affiliations:** 1 State Key Laboratory of Trauma, Burns and Combined Injury, Department of Army Occupational Disease, Research Institute of Surgery, Daping Hospital, Army Medical University, Chongqing, 400042 China; 2 Department of Orthopaedics, the First Affiliated Hospital of Zhengzhou University, Zhengzhou, 450042 Henan China; 3 State Key Laboratory of Trauma, Burns and Combined Injury, Research Department of Traumatic Shock and Blood Transfusion, Research Institute of Surgery, Daping Hospital, Army Medical University, Chongqing, 400042 China; 4 Department of Radiology, Chongqing No. 7 Hospital of Chongqing University of Technology, Chongqing, 400054 China

**Keywords:** Cytoskeleton, Mechanisms of disease, Molecular medicine, Neuroscience, Blood-brain barrier, Cell death in the nervous system, Cognitive neuroscience, Diseases of the nervous system, Learning and memory

## Abstract

Traumatic brain injury (TBI) is an important cause of death in young adults and children. Till now, the treatment of TBI in the short- and long-term complications is still a challenge. Our previous evidence implied aquaporin 4 (AQP4) and hypoxia inducible factor-1α (HIF-1α) might be potential targets for TBI. In this study, we explored the roles of AQP4 and HIF-1α on brain edema formation, neuronal damage and neurological functional deficits after TBI using the controlled cortical injury (CCI) model. The adult male Sprague Dawley rats were randomly divided into sham and TBI group, the latter group was further divided into neutralized-AQP4 antibody group, 2-methoxyestradiol (2-ME2) group, and their corresponding control, IgG and isotonic saline groups, respectively. Brain edema was examined by water content. Hippocampal neuronal injury was assessed by neuron loss and neuronal skeleton related protein expressions. Spatial learning and memory deficits were evaluated by Morris water maze test and memory-related proteins were detected by western blot. Our data showed that increased AQP4 protein level was closely correlated with severity of brain edema after TBI. Compared with that in the control group, both blockage of AQP4 with neutralized-AQP4 antibody and inhibition of HIF-1α with 2-ME2 for one-time treatment within 30–60 min post TBI significantly ameliorated brain edema on the 1st day post-TBI, and markedly alleviated hippocampal neuron loss and spatial learning and memory deficits on the 21st day post-TBI. In summary, our preliminary study revealed the short-term and long-term benefits of targeting HIF-1α-AQP4 axis after TBI, which may provide new clues for the selection of potential therapeutic targets for TBI in clinical practice.

## Introduction

Traumatic brain injury (TBI) is a major cause of death in young adults and children with high prevalence of long-term neurological functional deficits in survivors^1,2^. Brain edema is one of the most important pathophysiological characteristics of TBI and intractable clinical problem and has been proven to be an critical cause of intracranial hypertension and cerebral hernia, thereby leading to elevated morbidity and poor prognosis for TBI survivors^3–5^. The occurrence and development of traumatic brain edema is closely related to the dysfunction or structural damage of blood–brain barrier (BBB)^6,7^, and is disclosed to be linked with aquaporins (AQPs) by our studies and others^8–10^.

AQPs are a family of water channels distributed in the plasma membrane in endothelial cells and astrocytes, and play a key role in maintaining brain water homeostasis^11,12^. Among them, AQP4 is the most abundant aquaporin in the brain^13^. Despite of a water specific channel, AQP4 has been demonstrated to play various roles in neurotransmission, potassium transport, BBB integrity and so on^14–16^. Recently, accumulating evidences indicated that brain edema was closely associated with AQP4 in several cerebral diseases, and targeting AQP4 was responsible for tissue regulation of osmolarity and be beneficial for short-term and long-term outcomes, such as brain edema formation, neuronal damage and neurological functional deficits^9,17^. Although a positive relationship between the upregulated AQP4 protein expression and brain edema after TBI was revealed by our and other’s studies^10,18^, the effects of treatments by targeting the channel from short-term to long-term remains largely obscure.

Hypoxia-inducible factor-1 (HIF-1) is an essential transcription factor responsible for the induction of a series genes that facilitate adaptation under hypoxic conditions^19,20^. HIF-1 consists of a constitutively expressed β subunit and an oxygen-sensitive α subunit which is rapidly degraded in normoxia, whereas stabilized in hypoxia^21^. Similar to hypoxia, when TBI occurs, HIF-1α is prevented from being degraded, resulting in higher cellular level in 5 min which in turn leads to activation of the HIF-1 complex inducing the transcription of certain genes such as AQP4^22^. However, whether upstream suppression of HIF-1α could reduce AQP4 expression and thereby bring some short- and long-term benefits after TBI is still unclear.

The goal of this study was to explore the roles of AQP4 and its crucial upstream transcription factor HIF-1 on brain edema formation, neuronal damage and neurological functional deficits using CCI model, which might be helpful to seek new potential therapeutic targets for TBI.

## Methods

### Controlled cortical impact (CCI) injury model of rats

All animal procedures were performed according to protocols approved by the Laboratory Animal Welfare and Ethics Committee of the Army Medical University (Chongqing, China), and complied with the ARRIVE guidelines for the Care and Use of Laboratory Animals. The methods were carried out in accordance with the approved guidelines. Adult male Sprague Dawley rats weighing 220–260 g (g) were obtained from the Experimental Animal Center of the Research Institute of Surgery, Daping Hospital, Army Medical University, Chongqing, China. The animals were randomly divided into a sham group and TBI groups, the latter groups were further divided into neutralized-AQP4 antibody group, 2-ME2 group, and the corresponding IgG and isotonic saline control groups. The moderately controlled cortical impact model was established by modified Feeney method^23^ using a PinPoint craniocerebral injury impact device (Hatteras Instruments, Inc., Cary, NC, USA). After rats were anesthetized by intraperitoneal injection of pentobarbital sodium (40 mg/kg) and a circular bone window with a diameter of 5 mm was prepared, then subjected to impact tests. The impact parameters of brain tissue were as follows: diameter of impact head was 4.0 mm, impact depth was 4.0 mm, impact velocity was 2.5 m/s, impact time was 0.85 ms. Then, the bone window was immediately closed with bone wax. Rats in sham group performed the same surgical procedure except for impact.

### Inhibition of AQP4 and HIF-1α

In order to inhibit AQP4 and HIF-1α, TBI rats were intravenously injected via the tail vein with 0.25 ml isotonic saline containing AQP4 antibodies (1 μg/kg, Sigma Aldrich, USA, Cat#A5971) or 2-methoxyestradiol (2-ME2, 2.5 mg/kg of body weight, sigma Aldrich, USA, Cat#M5383-50MG) within 30–60 min after TBI, the corresponding controls were injected with IgG and isotonic saline, respectively. Molecular pathological detection and behavior tests were conducted on the 1st and 21st day following injury.

### Assessment of brain edema

Assessment of brain edema was performed as described as follows. Briefly, rats were anesthetized with intraperitoneal injection of 800 mg/kg pentobarbital sodium and sacrificed, the procedure is complied with American Veterinary Medical Association (AVMA) Guidelines for the Euthanasia of Animals, the whole brain was carefully harvested and immediately weighed the wet weight, then the dry weight was determined after drying for 72 h in heated oven at 80 °C. The brain water content is calculated as tissue water (%) = (wet weight-dry weight)/wet weight × 100.

### Western Blot assays

Brains were harvested and the hippocampal tissue beneath the injury side were excised for western blotting. Briefly, the total protein concentration was determined by Coomassie brilliant blue method. Western blot analysis was performed according to the standard procedures as previously described^10^. Samples were electrophoretic separated by sodium dodecyl sulfate (SDS)-polyacrylamide gel electrophoresis and transferred to the PVDF membrane. Then, the membranes were probed with the following primary antibodies overnight at 4 °C: anti-HIF-1α (1:1000, Cat. no. Ab216842, Abcam), anti-AQP4 (1:1000, Cat. no. ab128906, Abcam), anti-MAP2 (1:1000, Cat. no. Ab32454, Abcam), anti-SYN (1:1000, Cat. no. Ab32594, Abcam), anti-tau-5 (1:1000, Cat. no. Ab76128, Abcam), anti-pTau (ser404) (1:1000, Cat. no. Ab92676, Abcam), anti-MMP9 (1:1000, Cat. no. Ab76003, Abcam), anti-VEGFA (1:1000, Cat. no. Ab1316, Abcam), anti-occludin (1:1000, Cat. no. Ab240150, Abcam), anti-claudin-5 (1:1000, Cat. no. Ab131259, Abcam), anti-beta actin (1:10000, Cat. no. Ab4990, Abcam). After incubation with horseradish peroxidase-conjugated secondary antibodies, the membranes were visualized using chemiluminescence. Biotinylated protein ladder were used as an indicator to determine the protein molecular weight, and at least one fuller-length blot for each antibody was performed to confirm specific detection of the target antigen (Supplemental materials). The image analysis program (labwork 4.6, USA) was used to analyze the imprinted images.

### Immunofluorescence staining

Rats were intracardially perfused first with room temperature phosphate-buffered saline (PBS) then ice-cold 4% paraformaldehyde, post fixed overnight, and embedded in 30% sucrose until they sink. 25 µm sections were cut across the coronal plane, blocked with 5% BSA and permeabilized with 0.5% TritonX-100 in PBS for 30 min. Then, sections were incubated with dilutions of primary neuron-specific nuclear protein (NeuN) antibody (1:200, ab104224, Abcam) overnight at 4 °C, washed with PBS and incubated with Alexa Fluor 488-conjungated secondary antibody (1:300, Abcam, ab150113) for 1 h at room temperature. The sections were then washed and stained with DAPI for 15 min. Finally, the hippocampal CA1 NeuN-positive neuron numbers of the injured region in each group were calculated.

### Morris water maze test

The Morris water maze tests were performed to assess impairments in visual-spatial abilities (escape latency test) and visual short-term memory (spatial exploration experiment) in rats as previously described^10^. The escape latency and exploration time were investigated at pre-TBI, 7, 14 and 21 days post-TBI. In the water maze test, rats were placed in a large circular pool with a diameter of 120 cm and a height of 50 cm filled with opaque water maintained at 22.0 ± 1 °C and were given the task to swim to a platform that can be either visible or hidden. Before the experiment, decorate and mark the platform higher than the horizontal plane with a small flag. The rats were put into water and observed to see if they could swim to the platform freely and then the time of swimming to the platform were recorded. Rats with abnormal vision and motor dysfunction were excluded. Rats were first trained to find a randomly positioned visible platform in the swimming pool and were subjected to four trials per day for 3 consecutive days. For escape latency test, the escape platform with a diameter of 9 cm was placed in a constant position (in the center of one of the four quadrants) and 1.0 cm below the water surface. Time spent in finding the hidden platform was recorded as escape latency. If the rats fail to find the platform within 120 s, they were guided by the experimenter to the platform for 30 s, and the latency was recorded as 120 s. On the second day, the spatial exploration experiment was performed. After the hidden platform was removed, rats were placed at the same starting point and the searching time (residence time in the former target quadrant) in the quadrant of the original platform was recorded within 120 s. The time spent in finding the hidden platform (escape latency) and the searching time in spatial exploration experiment were measured and analysed from video recordings (Ethovision, Noldus Information Technology Inc, Leesburg, VA, USA).

### Statistical analysis

The data were presented as mean ± standard error (SEM) for all independent experiments. After the data were validated to meet normality by Shapiro-Wilks Test and Homogeneity of Variances by Levene’s Test, the comparisons among groups were analyzed by one-way analysis of variance (ANOVA), and the least significant difference (LSD) method were used for Post-hoc comparisons between groups. All data were analyzed using GraphPad Prism. *P* < 0.05 was defined as statistically significant.

## Results

### Blockage of AQP4 ameliorated brain edema in the acute phase after TBI

**Figure 1:**
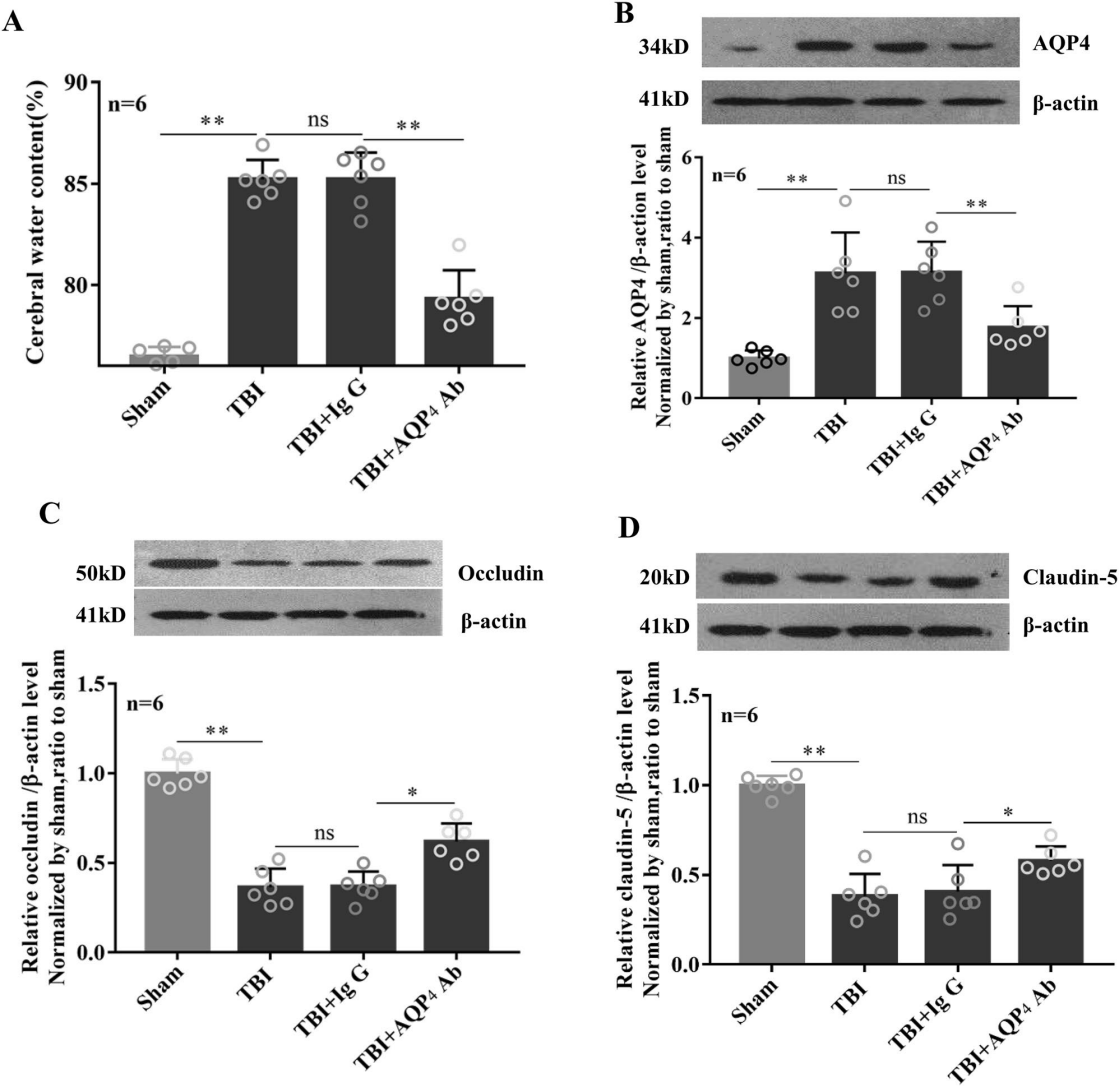
Blockage of AQP4 ameliorated brain edema in the acute phase after TBI. Rats were intravenously injected via the tail vein with 0.25 ml isotonic saline containing neutralized-AQP4 antibody (1 μg/kg of body weight) within 30–60 min after TBI, the control animals were injected with IgG. (**A**) On the 1st day post TBI, brain edema was examined by brain water content. Protein levels of AQP4 (**B**), Occludin (**C**) and Claudin-5 (**D**) were detected by western blot with β-actin as an internal control. The identical internal control β-actin blots were shared for Occludin and Claudin-5 blots because the detections were performed on the same blot membrane. Data are expressed as mean ± SEM, n = 6 per group. The comparisons among groups were analyzed by one-way ANOVA and the least significant difference (LSD) method. * *P* < 0.05, between the two groups. *ns* non-significant.

Following TBI, brain water content showed a dramatic increase, which was accompanied by elevated AQP4 protein level after 1 day, as compared to the sham group (Fig. 1A,B). Our previous study reported a positive correlation between the upregulated AQP4 protein level and the severity of brain edema^10^. To test the hypothesis that AQP4 is a potential drug target for TBI via aggravating the severity of brain edema, we intravenously injected neutralized-AQP4 antibody in rats within 30–60 min after TBI and investigated the acute outcome of brain edema formation. We found that animals in AQP4 antibody treatment group showed a significant reduction of 43.8% in AQP4 protein level in brains on the 1st day post-TBI (Fig. 1B), a decrease in brain water content (Fig. 1A), compared to that in IgG group, indicating that blockage of AQP4 ameliorates brain edema in the acute phase after TBI. Additionally, we detected the protein levels of two typical tight junction proteins closely related to vascular permeability, occludin and claudin-5, and found that both of which markedly decreased after TBI were effectively prevented with AQP4 antibody treatment, as compared to IgG group (Fig. 1C,D).

### Inhibition of HIF-1α reduced brain water content and improve blood–brain barrier function

**Figure 2:**
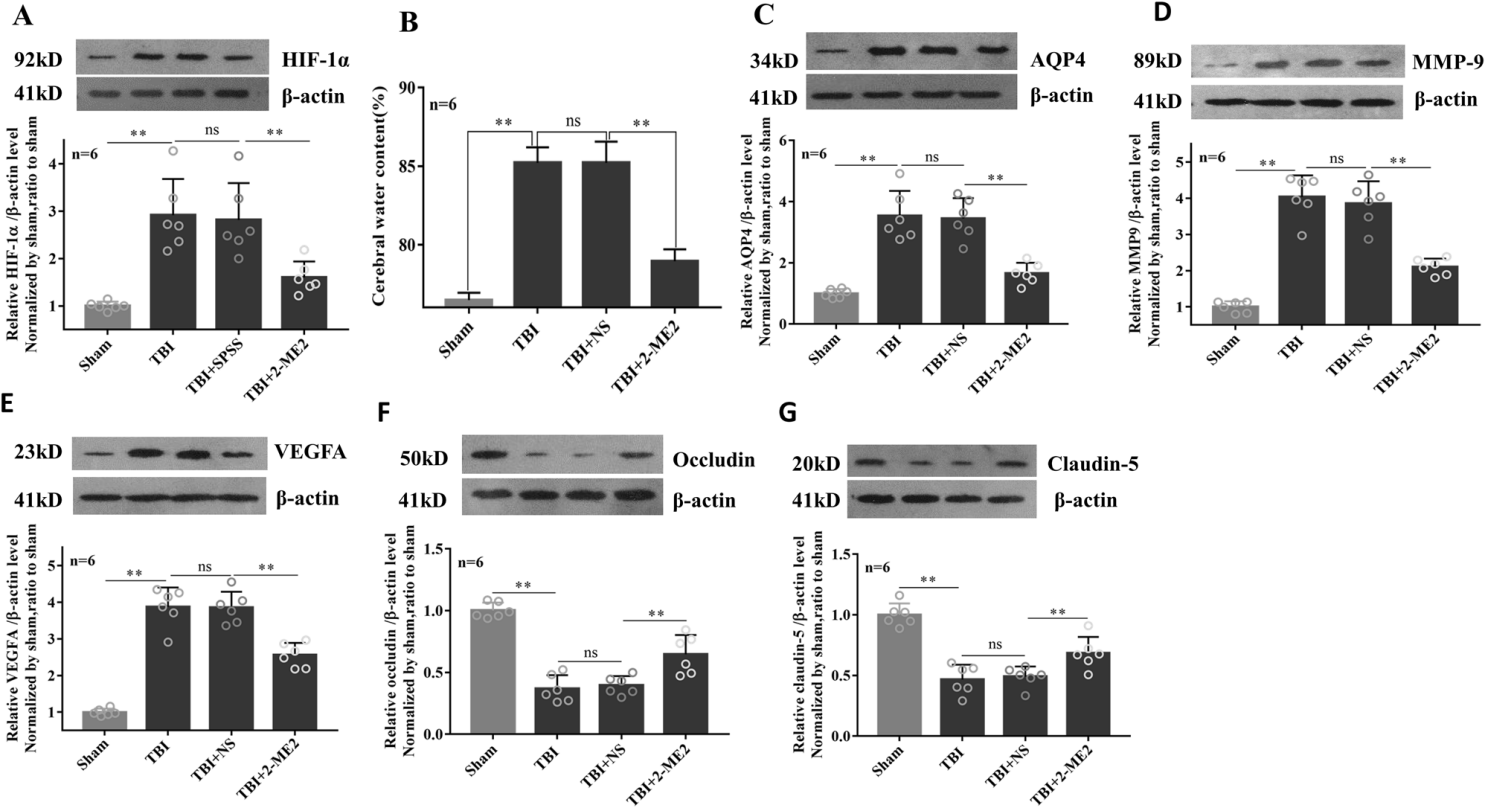
Inhibition of HIF-1α reduced brain water content and improve blood–brain barrier function. TBI rats were intravenously injected via the tail vein with 0.25 ml isotonic saline containing 2-ME2 (2.5 mg/kg of body weight) within 30–60 min after TBI, the corresponding controls were injected with isotonic saline. On the 1st day after injury, brain water content (**B**) was determined and protein levels of HIF-1α (**A**), AQP4 (**C**), MMP-9 (**D**), VEGF (**E**), tight junction protein levels of occluding (**F**) and claudin-5 (**G**) were detected by western blot. β-actin was used as loading controls for the total proteins. Data represent mean ± SEM (n = 6 per group). The comparisons among groups were analyzed by one-way ANOVA and the LSD method. Significant differences are shown by asterisks (*P < 0.05). *ns* non-significant.

Since HIF-1 has been identified as an important upstream transcription factor for AQP4 and considered to be a potential target for TBI according to our previous study^10^, we further assessed the involvement of HIF-1 in brain edema formation and BBB function. On the 1st day post-TBI, HIF-1α protein expression increased significantly, while it was effectively blunted with 2-ME2 treatment (Fig. 2A). Brain water content (Fig. 2B) and TBI-induced upregulation of AQP4 protein level (Fig. 2C) were markedly decreased after 2-ME2 treatment. We also investigated several protein expressions involved in regulating BBB permeability and observed that TBI-induced upregulation of both matrix metallopeptidase 9 (MMP-9) and vascular endothelial growth factor (VEGF) proteins were inhibited by 2-ME2 (Fig. 2D,E). Meanwhile, tight junction protein levels of occludin and claudin-5, which sharply decreased in TBI group, were significantly elevated on the 1st day post 2-ME2 treatment (Fig. 2F,G).

### Early post-traumatic inhibition of AQP4 and HIF-1α markedly alleviated TBI-induced neurological functional deficits in long term learning and memory

**Figure 3:**
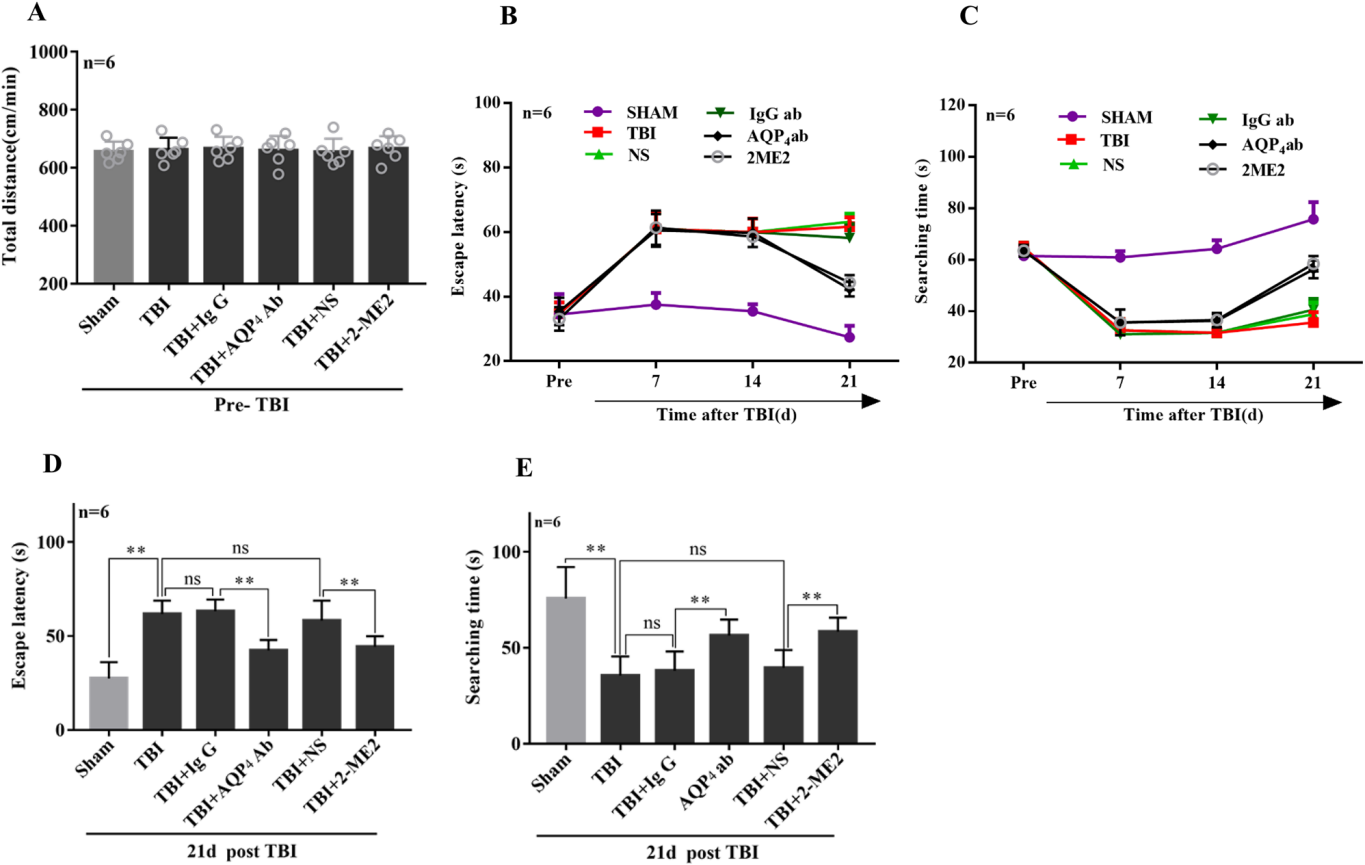
Early post-traumatic inhibition of AQP4 and HIF-1α markedly alleviated TBI-induced neurological functional deficits in long term learning and memory. In the water maze test, rats were placed in a large circular pool filled with opaque water and were given the task to swim to a platform that can be either visible or hidden. (**A**) Before TBI, the swimming distance per minute of rats in each group showed no significant difference**.** TBI rats were intravenously injected via the tail vein with 0.25 ml isotonic saline containing AQP4 antibodies (1 μg/kg of body weight) or 2-ME2 (2.5 mg/kg of body weight) within 30–60 min after impact, the corresponding controls were injected with IgG and isotonic saline, respectively. The Morris water maze tests were performed and the escape latency (**B**) and searching time (**C**) were investigated at the indicated time points, such as shown on the 21st day post-TBI in (**D**,**E**). Data represent mean ± SEM, n = 6 per group. The comparisons among groups were analyzed by one-way ANOVA with the LSD method. *P < 0.05, between the two groups. *ns* non-significant.

In order to investigate whether inhibition of AQP4 or HIF-1α within 30–60 min after experimental TBI could alleviate the long-term neurological functional deficits, cognitive behavioral analyses were assessed by Morris water maze tests. The time spent in finding the hidden platform (escape latency) and the searching time in spatial exploration experiment were measured at the indicated time points (Fig. 3B,C). Before TBI, the swimming distance per minute of rats in each group showed no significant difference (Fig. 3A). On the 21st day post-TBI, the latency time of rats in TBI group for completion of the maze task significantly increased and the searching time decreased, compared to that in sham group (Fig. 3D,E), indicating a declined cognitive behavioral performance of rats after TBI. Rats in neutralized AQP4 antibody treated group post-TBI showed a reduction in task completion time and an increase in searching time compared to that in IgG treated group (Fig. 3D,E). Animals in 2-ME2 treatment group showed similar results to AQP4 antibody treatment group (Fig. 3D,E). These results suggested an improvement of the long-term cognitive behavioral outcome by early post-traumatic inhibition of AQP4 or HIF-1α.

### Effects of AQP4 and HIF-1α inhibition on neuron loss and the expression levels of neuron structure-related proteins in hippocampus

**Figure 4:**
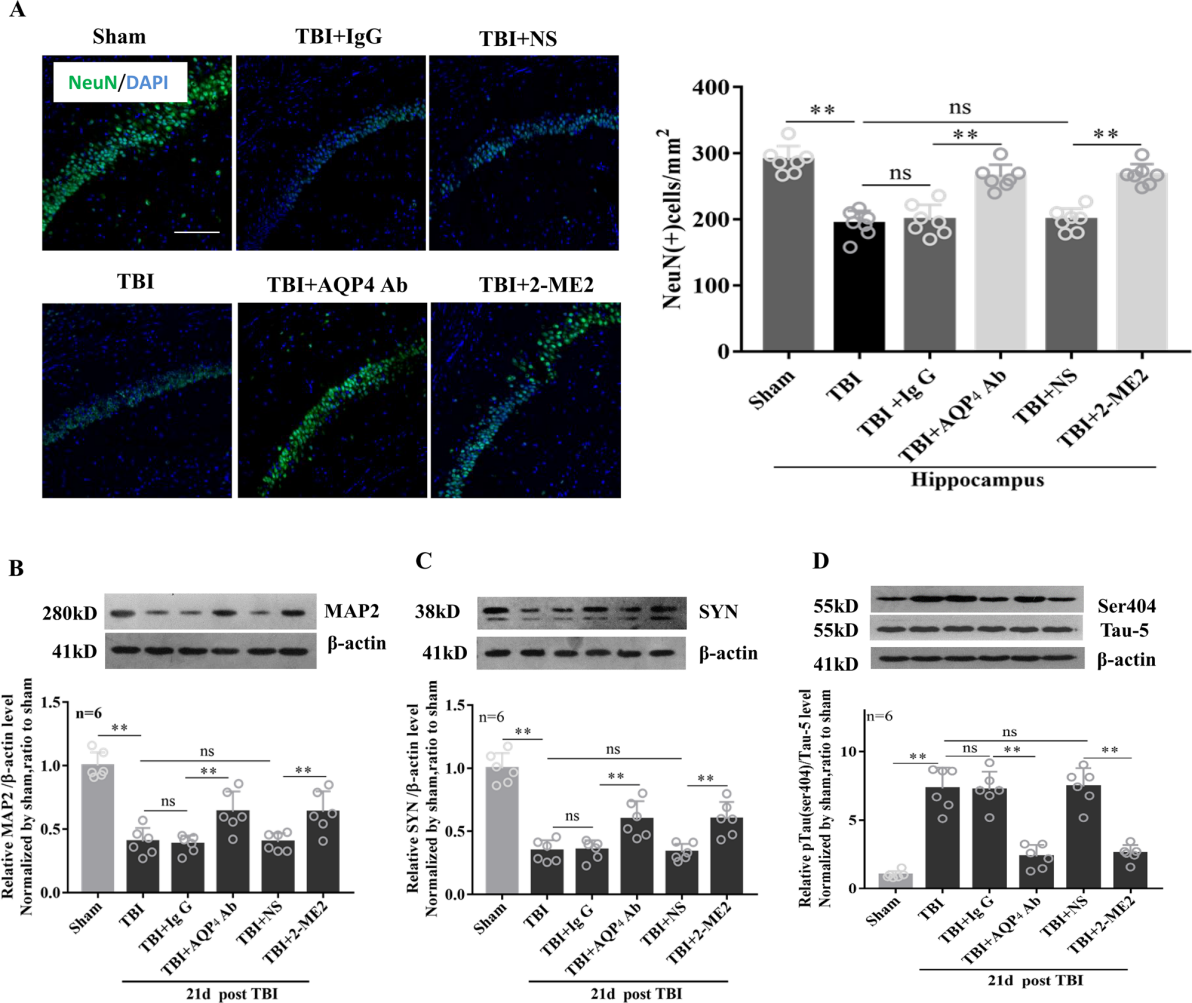
Effects of AQP4 and HIF-1α inhibition on neuron loss and the expression levels of neuron structure-related proteins in hippocampus. TBI rats were intravenously injected via the tail vein with 0.25 ml isotonic saline containing AQP4 antibodies (1 μg/kg of body weight) or 2-ME2 (2.5 mg/kg of body weight) within 30–60 min after impact, the corresponding controls were injected with IgG or isotonic saline, respectively. On the 21st day post-TBI, (**A**) the hippocampal CA1 NeuN-positive neuron numbers of the injured region in each group were calculated (scale bar 100 μm). The hippocampal protein levels of MAP2 (**B**) and SYN (**C**) were detected by western blot. (**D**) The protein expression of pTau (ser404) and Tau-5 was detected in each group. β-actin was used as loading controls for the total proteins. Data represent mean ± SEM, n = 6 per group. The comparisons among groups were analyzed by one-way ANOVA with the LSD method. * *P* < 0.05, between the two groups. *ns* non-significant.

To explore the potential molecular mechanism of cognitive behavioral improvement after targeting AQP4 and HIF-1α following TBI, we assessed the neuronal injury in hippocampus. On the 21st day post-TBI, the hippocampal CA1 NeuN-positive neuron numbers of the injured region showed a significant reduction, while it was markedly ameliorated by either blockage of AQP4 with neutralized-antibody or inhibition of HIF-1α with 2-ME2 (Fig. 4A). Next, we detected the expression levels of neuron structure-related proteins in hippocampus by western blot. The western blot analysis showed that the hippocampal protein levels of microtubule associated protein 2 (MAP2) and synaptophysin (SYN), which dramatically decreased on the 21st day post-TBI, were elevated after inhibition of either AQP4 or HIF-1α (Fig. 4B,C). In addition, we found that treatment with AQP4 blockage or HIF-1α inhibition markedly reduced the ratio of hippocampal pTau/Tau, which was elevated on the 21st day post-TBI (Fig. 4D).

## Discussion

In the present study, we demonstrated that the upregulation of AQP4 and HIF-1α contributes to brain edema formation, a critical process involved in intracranial hypertension and cerebral hernia following traumatic brain injury (TBI). Additionally, we found that inhibition of AQP4 and HIF-1α ameliorates neuronal damage and the long-term neurological functional deficits. Our data imply that the upregulated HIF-1α-AQP4 axis might be an important player in brain edema formation, neuronal damage and neurological functional deficits after TBI.

Although a number of factors are closely associated with the high morbidity and mortality of TBI, the development of brain edema remains the most notable predictor of outcome^5^. Recently, growing evidence showed that aquaporins, which belong to water channel family facilitating water transport, are important modulators for brain edema in the development of several brain diseases^24–27^. Of these, AQP4, the most abundant aquaporin in the brain, has been reported to involve in brain edema and has potentially important clinical implication by stroke and ischemia research^28–30^. In brain tissue, AQP4 is mainly localized to astrocytes and endothelium and forms integral components of the blood brain barrier (BBB) and is thought to be responsible for tissue regulation of homeostasis and osmolarity^13^. In a closed-head diffuse TBI model, Shenaq et al.^9^ reported that blockage of AQP4 with antibodies is beneficial for blunting brain edema formation. Using a CCI injury model of rats, we found that brain water content was attenuated following the administration of AQP4 antibodies. Since brain edema is characterized by the accumulation of water content within the brain parenchyma, our data suggested that blockage of AQP4 with neutralized antibodies effectively ameliorates the development of brain edema after TBI.

Previous studies have reported that the occurrence and development of brain edema mutually interacted with the abnormal structure and function of neurons, thereby led to the long-term neurological deficits^31,32^. Recently, AQP4 has been reported to play important roles in brain edema formation at the early stage of several brain diseases, and thereby participates in the secondary neuronal damage and brain dysfunction at the later stage. For instance, in focal and global cerebral ischemia models, AQP4 knockout mice showed reduced cerebral edema, infarct volume, intracranial pressure and neuronal loss *vs* control mice^17^. Wallisch^33^ reported that pharmacological AQP4 inhibition significantly reduced pediatric asphyxial CA-related cerebral edema and improved neurobehavioral outcomes. In this study, we found that AQP4 inhibition with antibody reduced the loss of hippocampal neurons, but also resulted in shortened task completion time and prolonged exploration time on the 21st day after TBI. Our data provided evidence that early post-TBI AQP4 inhibition ameliorates brain edema and the development of secondary neuronal damage and neurological functional deficits.

Since HIF-1 has been identified to be a vital upstream transcription factor for AQP4, we further examined the roles of 2-ME2, a BBB-permeable estradiol metabolite potently inhibits the active α subunit of HIF-1, on the short- and long-term complications following TBI. On the 1st day post-TBI, the severity of brain edema was attenuated with early 2-ME2 administration after impact, as evidenced by reduction in brain water content. Accumulated evidence has linked the dysregulation of proteins such as AQP4, MMP-9 and VEGF to the development of BBB hyperpermeability after TBI^8,34,35^. Our data showed that TBI-induced upregulation of AQP4, MMP-9 and VEGF proteins were attenuated by 2-ME2. Meanwhile, tight junction proteins, such as occludin and claudin-5, which sharply decreased in TBI group, were significantly suppressed after 2-ME2 treatment.

Although several previous studies used 2ME2 in the context of TBI and other types of acute brain injuries, they mainly explored the roles of HIF1α in brain edema formation, BBB disruption, and neuroprotection at the early stages of brain injury. For example, In the rat subarachnoid hemorrhage model^36^, treatment with 2ME2 suppressed the expression of HIF1α, BNIP3 and VEGF and reduced cell apoptosis, BBB permeability, brain edema, and neurologic scores. In Rice-Vannucci model of neonatal hypoxic ischemic brain injury^37^, 2ME2 exhibited dose-dependent neuroprotection by decreasing infarct volume and reducing brain edema at 48 h post hypoxic ischemic brain injury. In the mice controlled cortical impact model^38^, intraperitoneal 2ME2 administration 30 min after TBI caused a dose-dependent reduction in secondary brain damage after 24 h. Using the rat Marmarou closed head force impact model, some previous researches^8,22^ reported the HIF1α signaling in AQP4 upregulation after TBI, these data also suggested that HIF1α plays a role in brain edema formation and BBB disruption via a molecular pathway involving AQP4.

Up to now, evidence of treatments of targeting HIF1α-AQP4 axis after TBI in an attempt to improve neurological outcome is largely obscure. Despite Shenaq et al.^9^ provided some research evidence in a closed-head diffuse TBI model, it is different from the CCI model applied in our study. In this study, we found that rats in 2-ME2 treated group showed a decrease in the loss of hippocampal neurons, a reduction in task completion time and an increase in exploration time on the 21st day post-TBI, compared to that in control group. Therefore, these results demonstrated that early post-traumatic administration of 2-ME2 improves BBB integrity in short and long term after TBI. Based on the role of targeted AQP4 in ameliorating the complications of TBI together with the evidence that AQP4 is identified as a target gene of HIF1, our data provided in vivo clues for the importance of HIF-1α-AQP4 axis in BBB dysfunction and the secondary brain damage following TBI.

Hippocampus is known to be important for numerous neurological functions, such as long term learning and memory. Previous evidence has revealed that the neurocognitive deficits caused by TBI are closely related to the abnormal expression and protein modification of neuronal cytoskeleton proteins such as microtubule associated protein 2 (MAP2) and synaptophysin (SYN) in the hippocampus^39,40^. We found that the hippocampal protein levels of MAP2 and SYN, which significantly decreased on the 21st day post-TBI, were markedly elevated after inhibition of either AQP4 or HIF-1α. In addition, we detected Tau protein phosphorylation at ser^404^ site, which is the main component of neurofibrillar tangles and is causally related to memory loss in brain-related diseases, such as Alzheimer's disease (AD), aging and TBI^41–43^. As shown in Fig. 4, the ratio of hippocampal pTau/Tau in TBI rats dramatically increased compared with that in sham group, while it was significantly reduced after inhibition of either AQP4 or HIF-1α. These findings implied that inhibition of AQP4 and HIF-1α protects the long-term neurological function, which might be correlated with the alleviated hippocampal neuronal skeleton and synaptic damage.

In conclusion, our results showed that early post-TBI AQP4 and HIF-1α inhibition blocks the development of brain edema formation, neuronal damage and neurological functional deficits. Although our understanding of the underlying mechanisms is limited, our data indicated the importance of the upregulated HIF-1α-AQP4 axis in the short- and long-term complications following TBI. This study provides in vivo evidence and raises a possibility for targeting HIF-1α-AQP4 axis as a therapeutic strategy for treating brain edema and long-term neurological functional deficits following TBI.

## Supplementary Information

Supplementary Information.

## Abbreviations

AD: Alzheimer's disease
AQP4: Aquaporin-4
BBB: Blood–brain barrier
CCI: Controlled cortical injury
HIF-1α: Hypoxia inducible factor-1α
MAP2: Microtubule associated protein 2
MMP-9: Matrix metallopeptidase 9
NeuN: Neuron-specific nuclear protein
SDS: Sodium dodecyl sulfate
SYN: Synaptophysin
TBI: Traumatic brain injury
VEGF: Vascular endothelial growth factor
2-ME2: 2-Methoxyestradiol
