# Supplementary material for: Inhibition of HIF-1α-AQP4 axis ameliorates brain edema and neurological functional deficits in a rat controlled cortical injury (CCI) model
Source: Sci Rep. 2022 Feb 17;12:2701. doi: 10.1038/s41598-022-06773-9 (PMC8854620; doi:10.1038/s41598-022-06773-9)
Supplement: Supplementary file 1 — Supplementary Information. [file 41598_2022_6773_MOESM1_ESM.docx]

**Supplemental Figures:**

1. **AQP4 protein expression**

**A1. A2.**


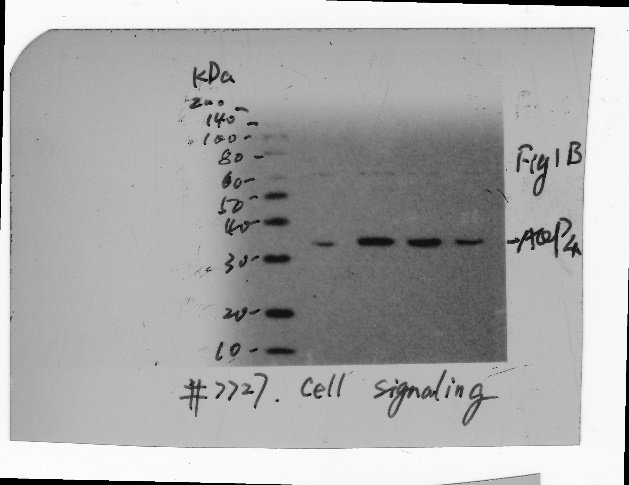

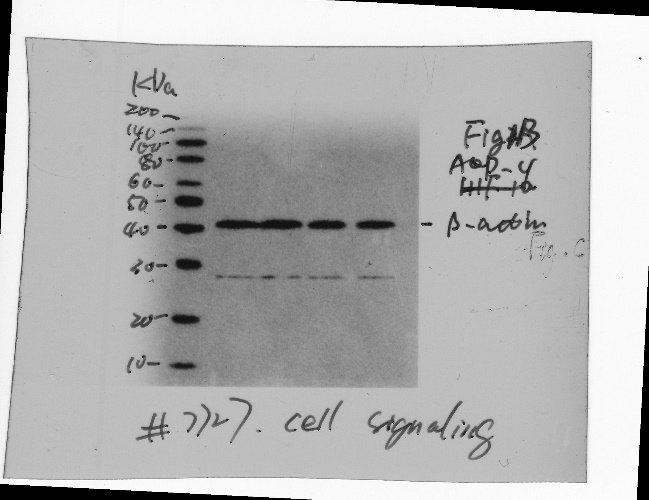


**A3. A4.**


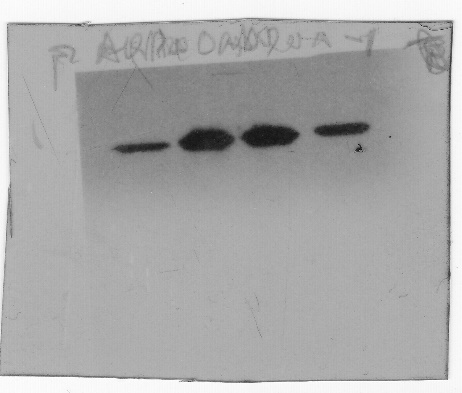

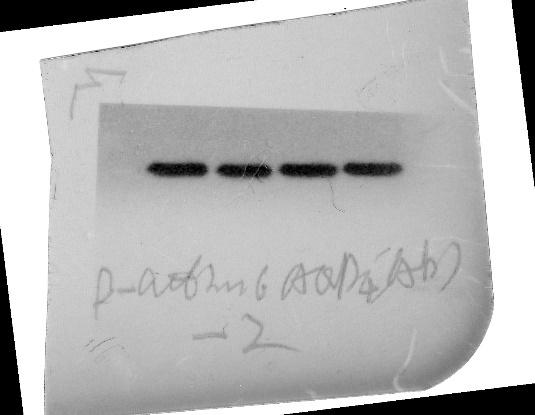


1. **Occludin protein expression**

**B1. B2.**




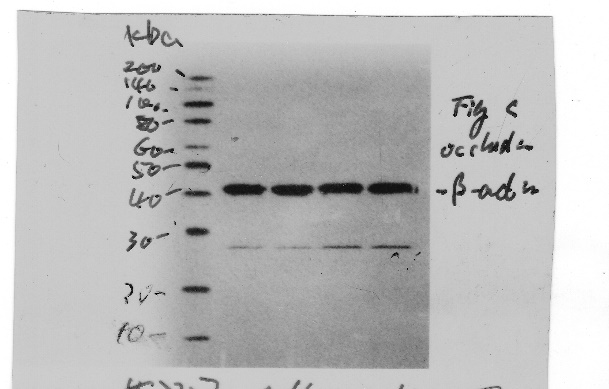


**B3. B4.**


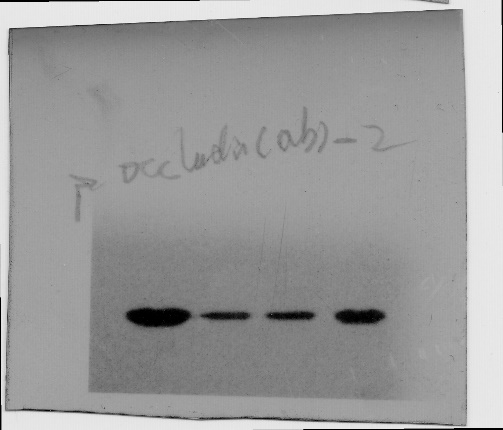

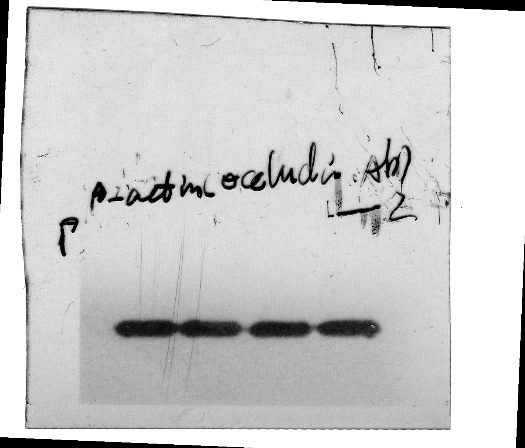


1. **Claudin-5 protein expression**

**C1. C2.**


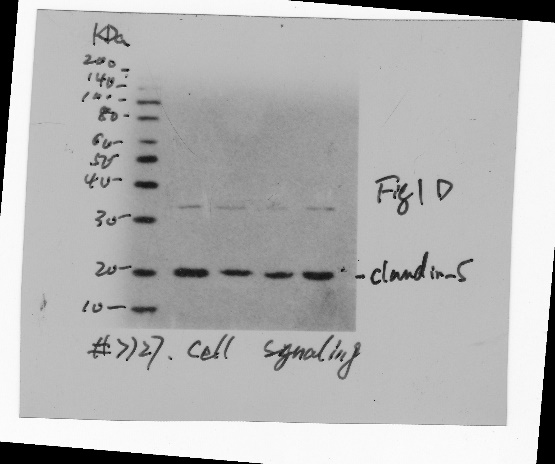

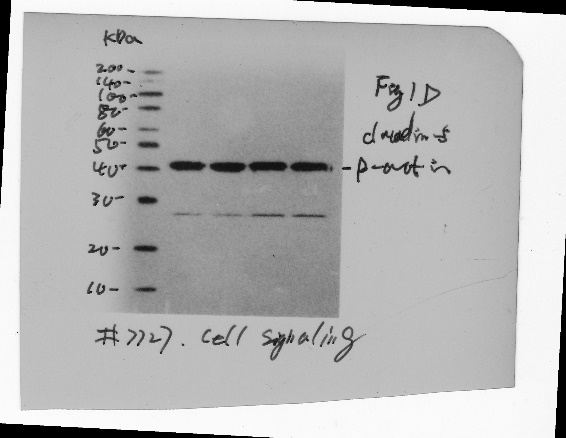


**C3. C4.**




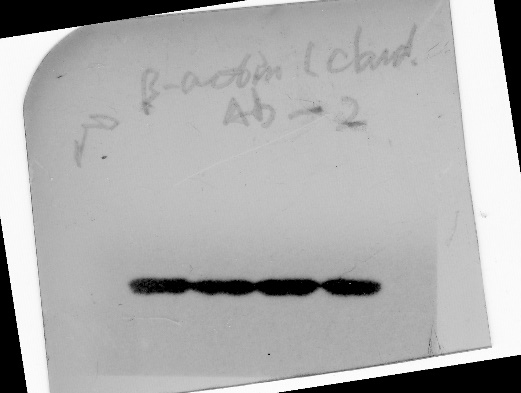


**Figure S1. Protein levels of AQP4 (A), occluding (B) and claudin-5 (C) were detected by western blot with β-actin as an internal control.**

1. **HIF-1α protein expression**

**A1. A2.**


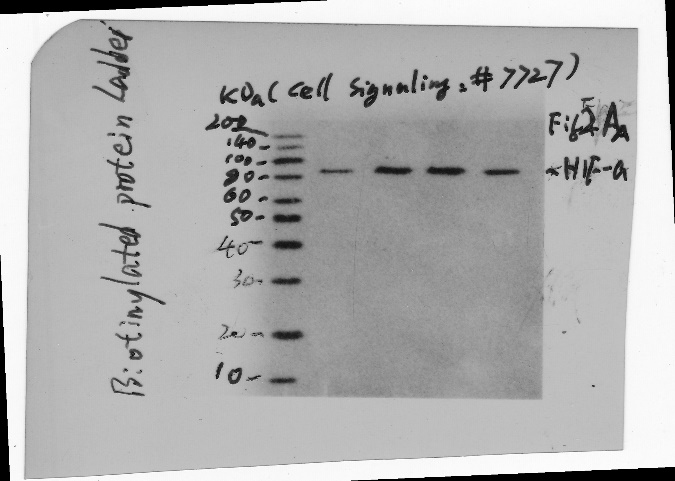

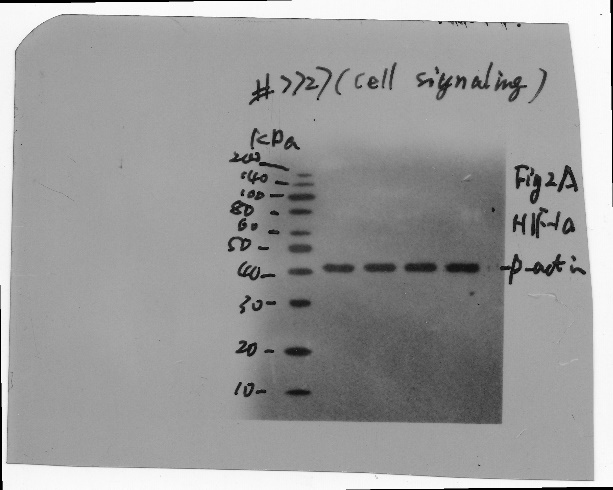


**A3. A 4.**




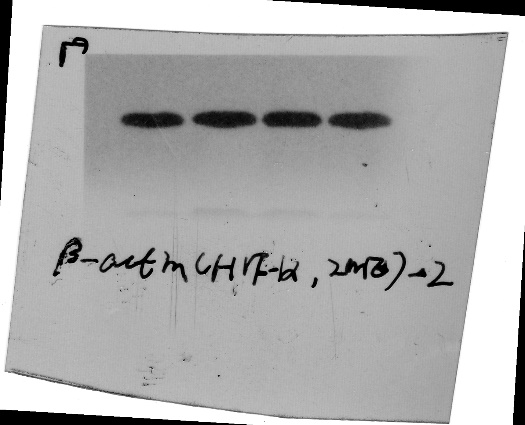


1. **AQP4 protein expression**

**B1. B2.**


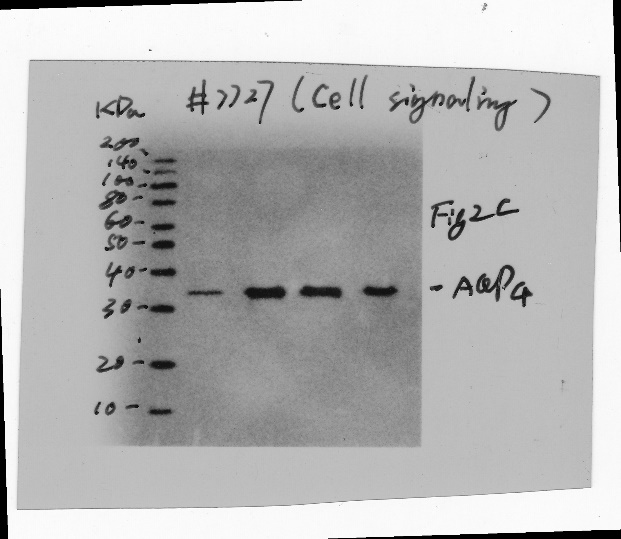

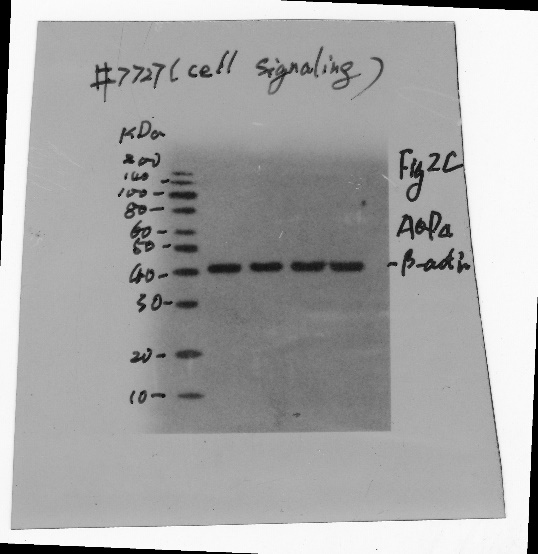


**B3. B4.**




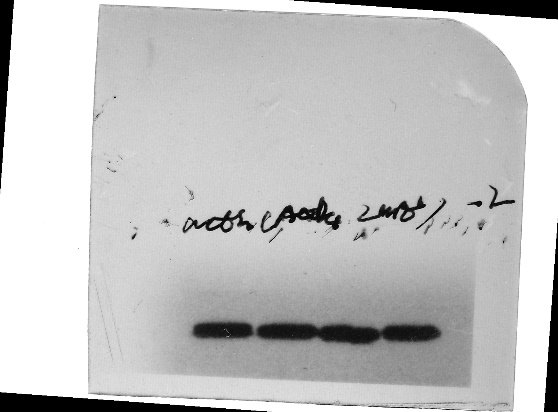


1. **MMP-9 protein expression**

**C1. C2.**


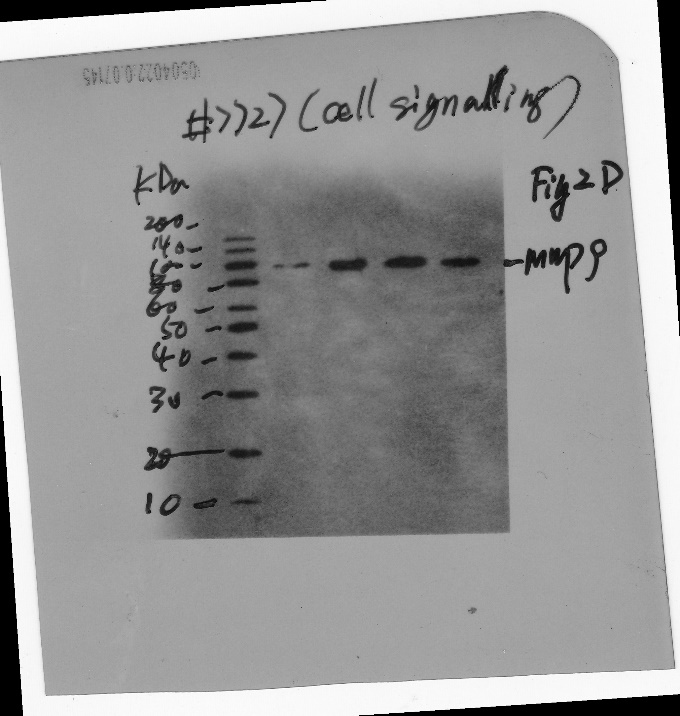

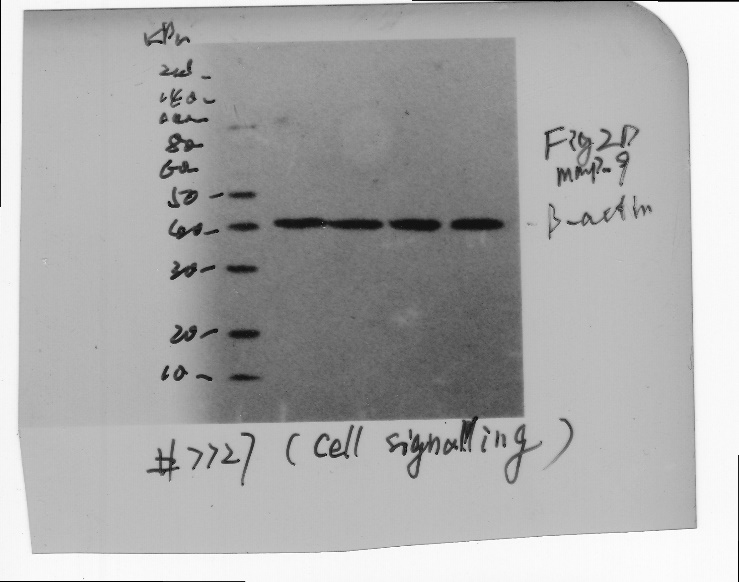


**C3. C4.**


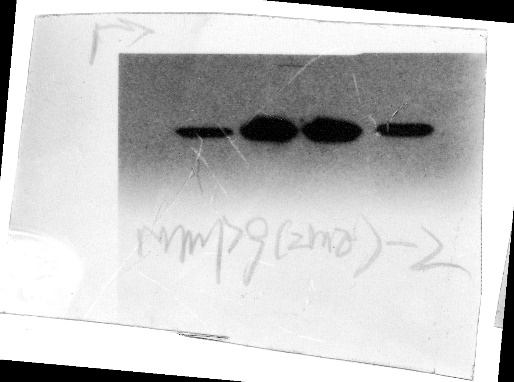

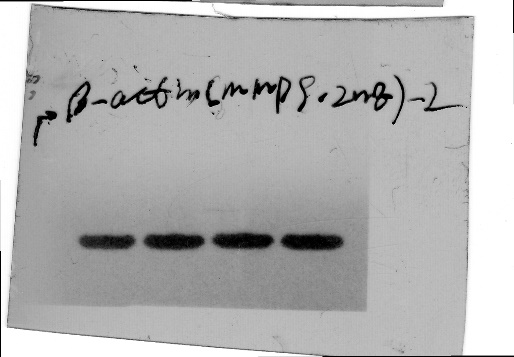


1. **VEGF protein expression**

**D1. D2.**


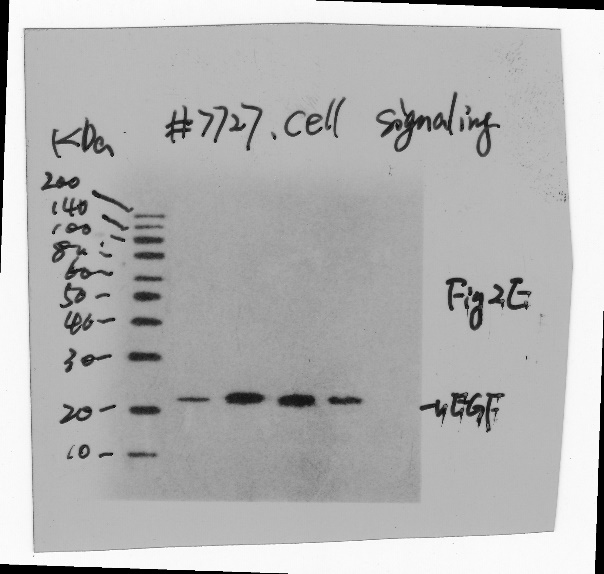

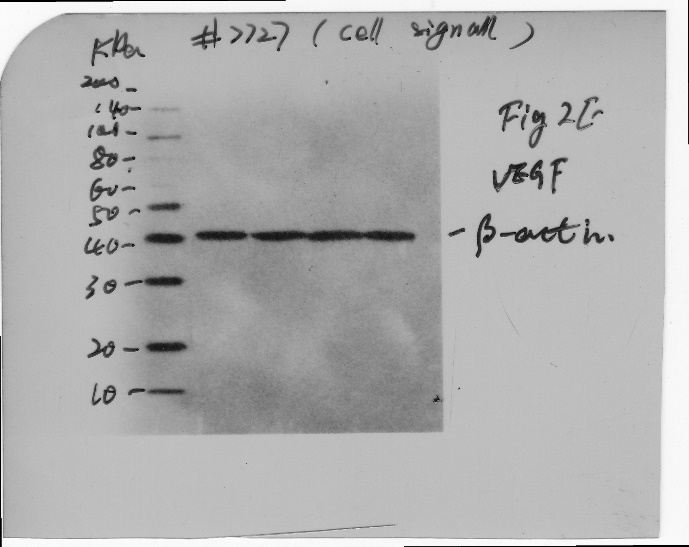


**D3. D4.**


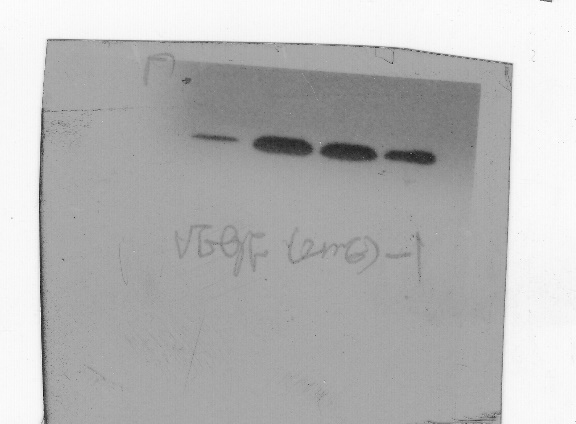

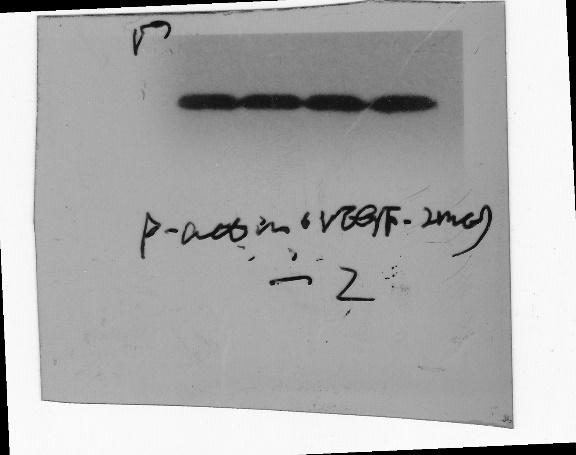


1. **Occludin protein expression**

**E1. E2.**


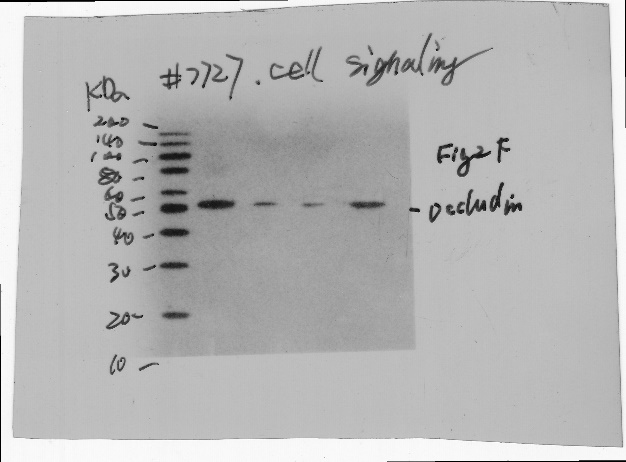

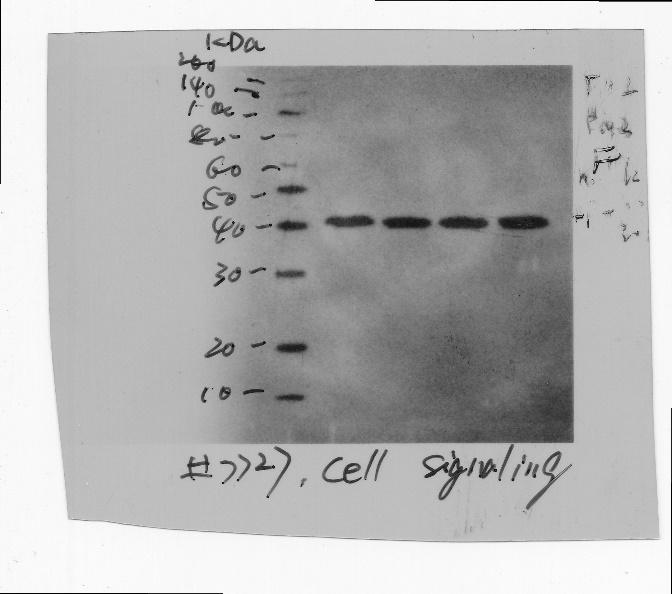


**E3. E4.**


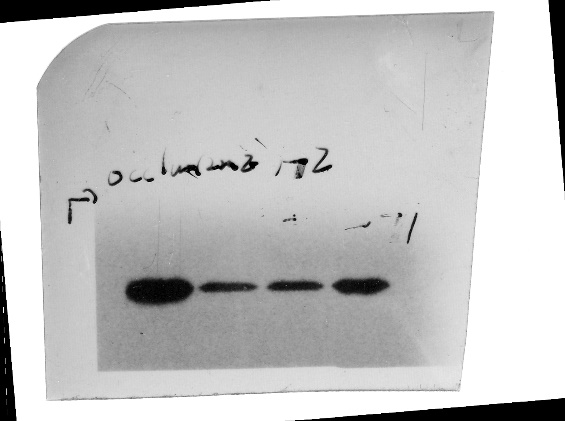

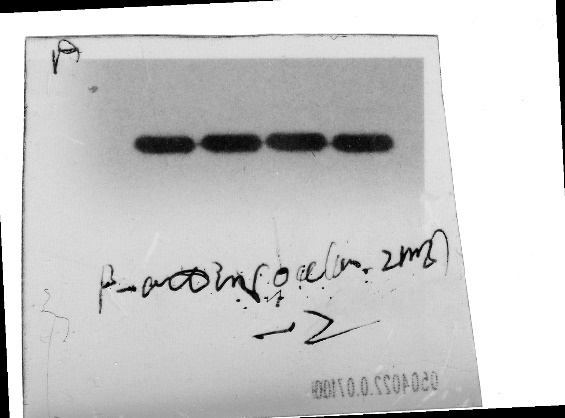


1. **Claudin protein expression**

**F1. F2.**


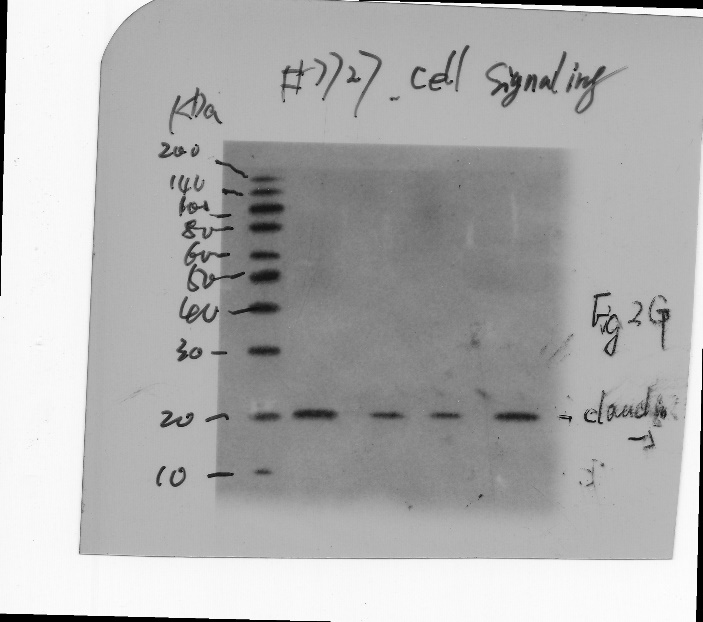

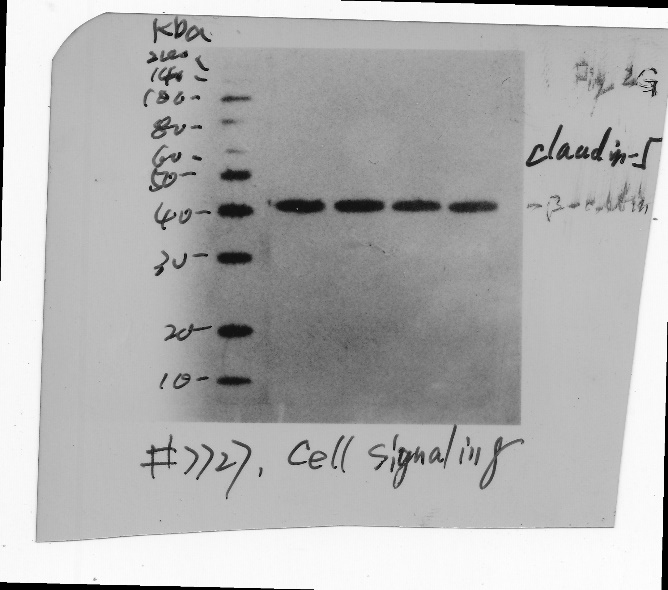


**F3. F4.**


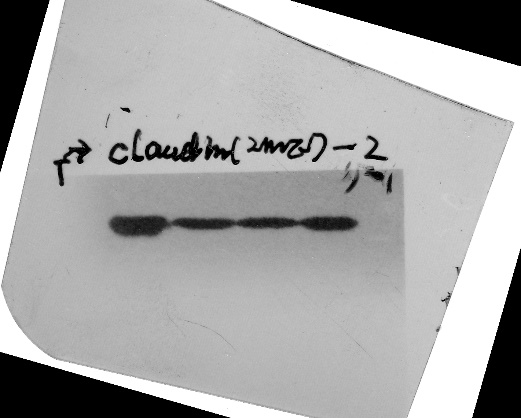

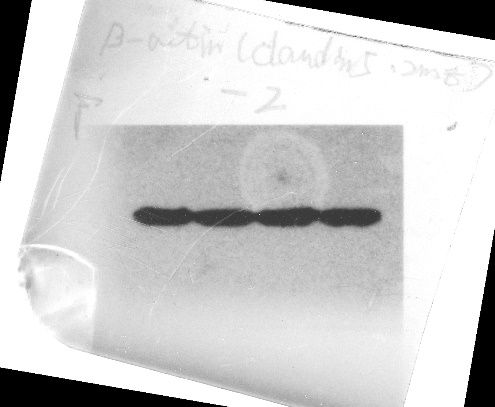


**Figure S2. Protein levels of** **HIF-1α (A), AQP4 (B)****，****MMP-9(C), VEGF(D),occluding (E) and claudin-5 (F) were detected by western blot with β-actin as an internal control.**

1. **MAP2 protein expression**

**A1. A2.**


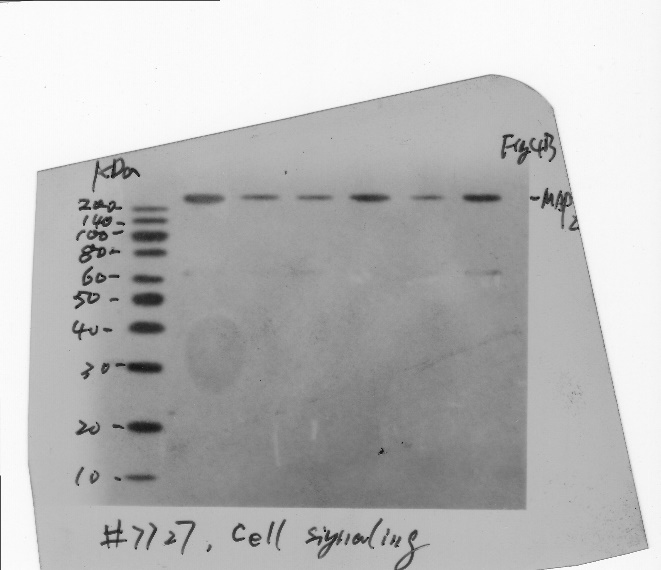

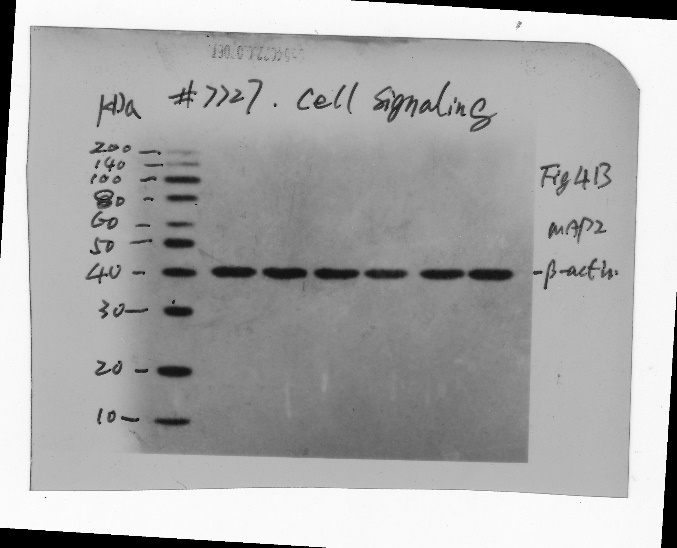


**A3. A4.**







1. **SYN protein expression**

**B1. B2.**


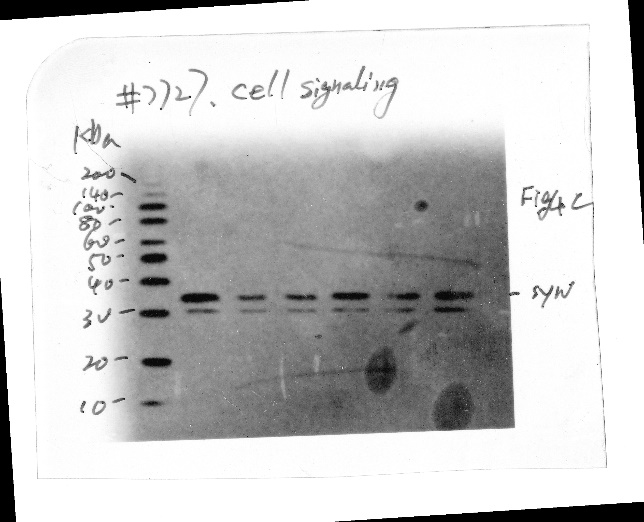

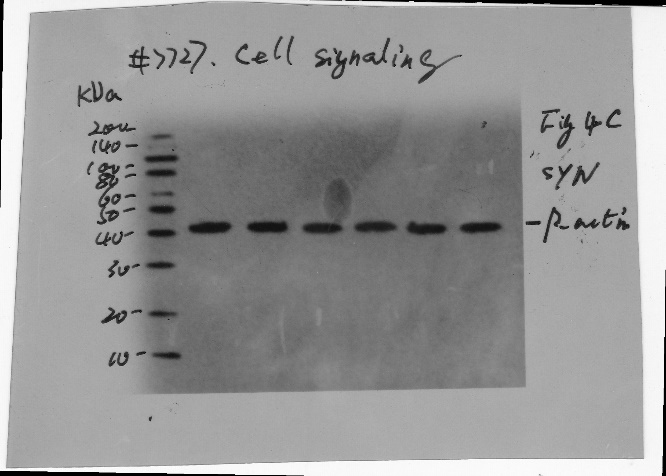


**B3. B4.**


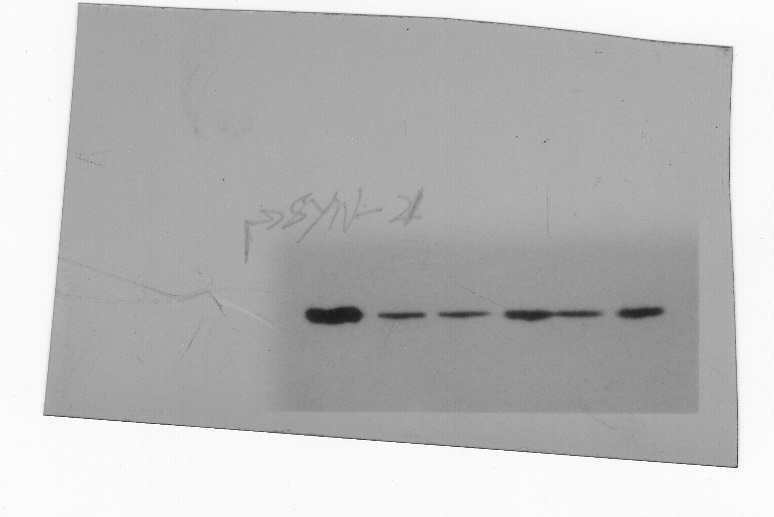




1. **Ser404 and Tau-5 protein expression**

**C1.**


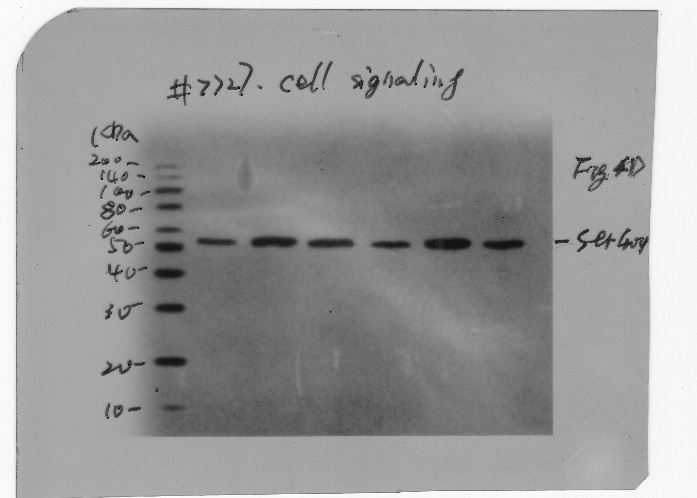

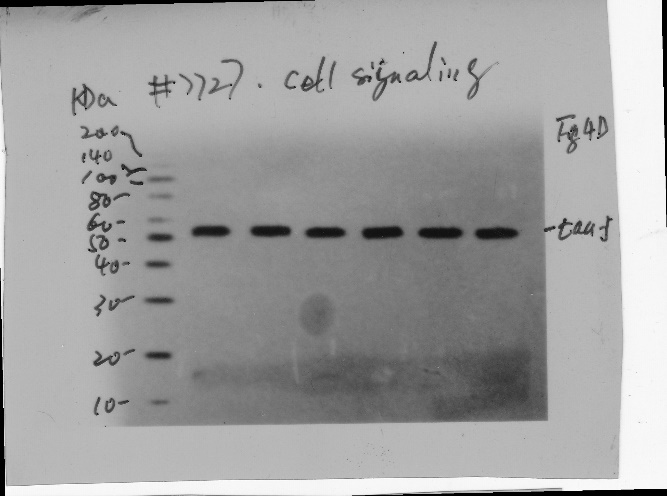


**C2.**


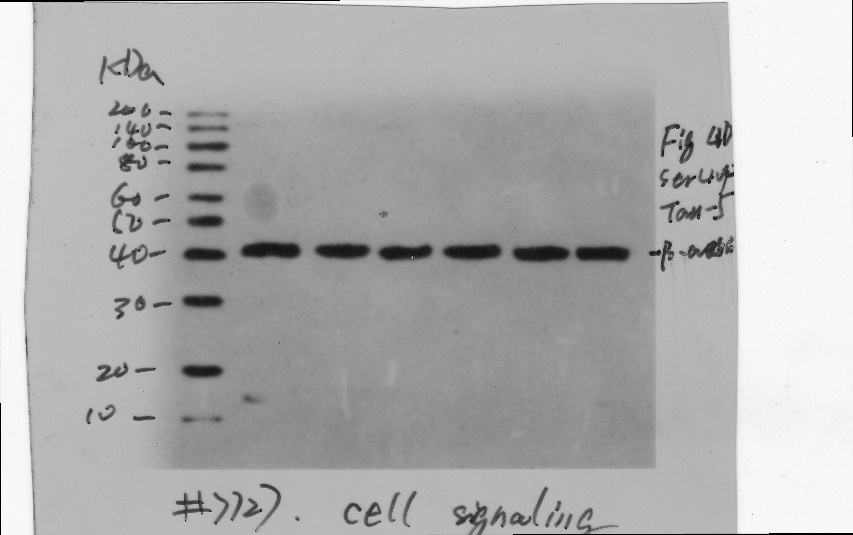


**C3.**


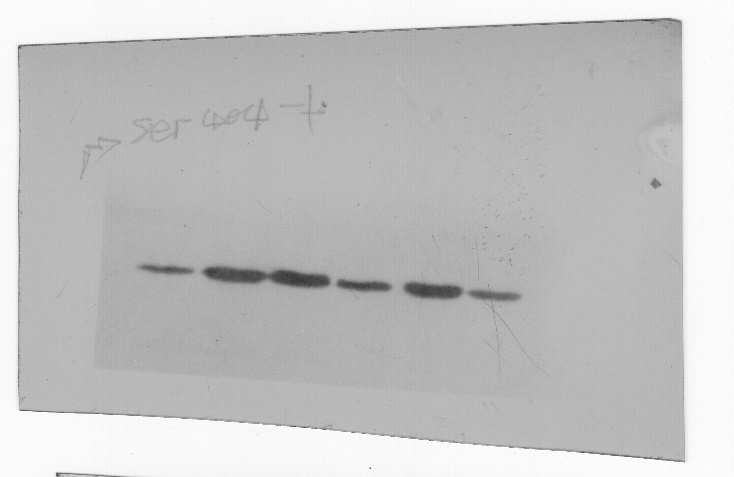

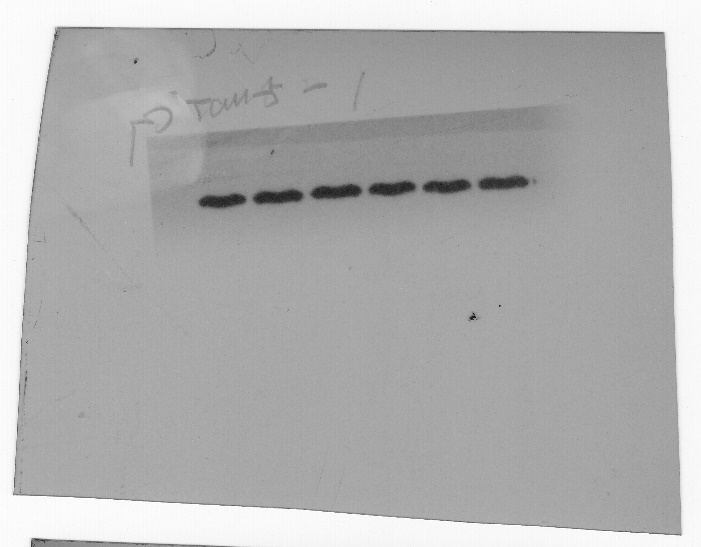


**C4.**


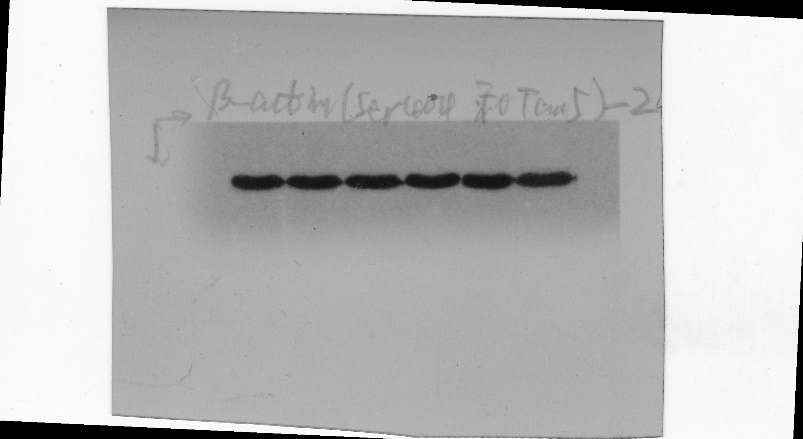


**Figure S3. Protein levels of MAP2 (A), SYN (B), Ser404(C) and Tau-5 (D) were detected by western blot with β-actin as an internal control.**
